# Integrated assessment of irrigation and agriculture management challenges in Nepal: An interdisciplinary perspective

**DOI:** 10.1016/j.heliyon.2024.e29407

**Published:** 2024-04-10

**Authors:** Santosh Nepal, Nilhari Neupane, Sanju Koirala, Jonathan Lautze, Ram Narayan Shrestha, Dinesh Bhatt, Nirman Shrestha, Manju Adhikari, Santosh Kaini, Shanta Karki, Jigyasha Rai Yangkhurung, Kapil Gnawali, Ananta Man Singh Pradhan, Krishna Timsina, Saurav Pradhananga, Manohara Khadka

**Affiliations:** a International Water Management Institute, Nepal Office, Kathmandu, Nepal; b International Water Management Institute, South Africa Office, Pretoria, South Africa; c Department of Water Resources and Irrigation, Ministry of Energy, Water Resources and Irrigation, Lalitpur, Nepal; d Water Resources Research and Development Centre, Ministry of Energy, Water Resources and Irrigation, Kathmandu, Nepal; e Department of Agriculture, Ministry of Agriculture and Livestock Development, Kathmandu, Nepal; f Water and Energy Commission Secretariat, Kathmandu, Nepal; g National Agricultural Policy Research Centre (NAPREC), Nepal Agricultural Research Council (NARC), Lalitpur, Nepal

**Keywords:** agriculture water, gender and governance, socioeconomic and market, Holistic assessment, Western Nepal, Likert scale

## Abstract

Agriculture plays a critical role in ensuring food and nutrition security, livelihood, and rural employment in Nepal. Despite substantial investments and institutional reforms, irrigation projects have faced consistently low performance. While existing studies have shed light on technical aspects of irrigation performance, they often focus on specific themes rather than holistic evaluations of sustainability. This research systematically assesses barriers and challenges to effective irrigation water management in Nepal by assessing and ranking the challenges faced by three irrigation systems in western Nepal: Mahakali, Rani Jamara Kulariya, and Babai. To investigate these challenges, we collected data from 449 households, which provided insights into 33 indicators representing key barriers to effective irrigation and agricultural management. The identified challenges were categorized into four broad thematic areas: physical and structural, agricultural and water, socioeconomic and market, and gender and governance. A comprehensive evaluation was conducted to compare these challenges among the three irrigation schemes, different thematic areas, and various locations within each scheme (namely, the head, mid, and tail sections of the system). The findings revealed that timely access and availability of fertilizers, spring water availability and fair market prices of agricultural products are the most significant challenges. The Babai irrigation system faced the most substantial challenges among the three systems, particularly in the mid section. These findings emphasize the interconnectedness of these challenges, highlighting the need for a holistic approach to planning, implementation, and management. Integrated strategies are essential to address socioeconomic, market, and endogenous farming issues, ensuring reliable irrigation water availability for sustainable agricultural production.

## Highlights

• A novel approach capturing interdisciplinary perspectives on irrigation and agriculture management challenges was developed.
• Timely access and availability of fertilizer emerged as the critical barriers to enhancing agricultural productivity.
• These challenges are intricately connected and predominantly stem from the lack of consistent year-round irrigation.

## 1 Introduction

Agriculture is crucial in ensuring food and nutrition security and improving livelihoods. Globally, agriculture is the largest water-consuming sector, accounting for 87 % of total water consumption. Irrigation alone accounts for around 60 % of the world's freshwater withdrawals [1]. In Nepal, 98 % of total freshwater withdrawals are used in the agricultural sector [2]. Twelve major irrigation development projects have been implemented in Nepal since 1990, at a total cost of more than NPR 67 billion (approx. 800 million USD at 2019 prices) and a combined irrigation area of approximately 570,000 ha [3]. As per the irrigation master plan 2019, irrigation infrastructure has been developed in nearly 50 % of Nepal's 2.5 million hectares (ha) of potentially irrigable land. There remains a need to expand current irrigated areas, improve the efficiency of existing systems and improve water productivity for food security [[4], [5], [6],[7]].

Unfortunately, despite decades of investment and institutional improvement, Nepal's irrigation projects exhibit low performance [8]. Indeed, when comparing Nepal's crop yields from irrigated agriculture with those of other South Asian countries, there appears to be room for improvement. For example, the average yield of rice and wheat is 3.8 t ha^−1^ and 3 t ha^−1^ respectively in Nepal [9] whereas Bangladesh, India and China have 4.8 t ha^−1^, 4.2 t ha^−1^, and 7.1 t ha^−1^ rice yields respectively in 2021 whereas wheat yield was 3.2 t ha^−1^, 3.4 t ha^−1^, and 5.8 t ha^−1^ for the same year [10]. Besides water, production inputs such as access to capital, credit, fertilizer, labour availability, governance, institutions, and markets are important for improving water and overall agricultural productivity at the farm level [11,12].

Constraints to irrigation and agricultural performance are typically attributed to one or more of four factors [[13], [14], [15], [16], [17]].

1. Inadequate and/or suboptimal physical infrastructure
2. Water shortage for agriculture
3. Socio-economic and marketing factors
4. Gender equality, social inclusion, and governance issues

The first constraint concerns the physical infrastructure of irrigation systems including access to canal water, canal seepage, water logging, sedimentation and flooding. Promoting advanced technologies to improve ‘crop per drop’ has long been discussed. Flooding has remained a major problem annually in irrigation systems [18,19]. Canal leakage, water logging and sedimentation are major problems in Nepalese irrigation systems. Most irrigation systems are run-of-river types characterized by high sediment loads and insufficient regular maintenance, making them unreliable and inadequate in terms of water delivery [20].

The second constraint is the challenges around agricultural water management including issues like year-round availability of irrigation water, irrigation technologies, technical support to farmers, fertilizer, and seeds [[16], [21], [22]]. In Nepal, during the dry season, the mid and tail sections of the canal system experience water shortage particularly within the surface irrigation network. This is a common problem in the agency-managed irrigation system, where the command area is continuously expanding and irrigation infrastructure is under construction [23]. For example, in the Babai irrigation scheme, irrigation infrastructure covers 25,000 ha command area, whereas Adhikari et al. [24] suggested that the canal water is sufficient for only 6,300 ha in winter and 4,000 ha in spring. Appropriate water use is essential to improve productivity from irrigated agriculture, along with the timely provision of appropriate fertilizer, improved seeds, and other agricultural inputs [3,25,26].

The third constraint concerns the socio-economic aspect encompassing factors such as market, and access to agricultural inputs like fertilizers and seeds**.** There is a need to improve water productivity by addressing the salient socio-economic challenges such as access to capital, quality seed, credit, input subsidies, fertilizer availability, markets [[27], [28], [29], [30], [31],[32]], storage facilities, labour shortage, operation and maintenance costs and associated conflicts [15,33,34]. Lack of access to capital by farmers reduces water productivity [35,36] by limiting farmers' investment in production inputs like fertilizers, high-yielding varieties (HYVs), labour, energy, and technologies [37]. The pivotal role in crop production is played by timely access to improved seeds and fertilizers; nevertheless, obtaining fertilizer on time has become a recurrent issue in Nepal in recent years [38]. In many surface irrigation schemes in Nepal, an acute shortage of labour due to increasing labour migration has been impacting the operation and maintenance of the canals resulting in declines in both irrigation efficiency and command area and increased water-related conflicts directly or indirectly compromising the overall water productivity of the system [39].

The fourth constraint is around factors related to gender, social inclusion, and governance. The deep-rooted patriarchal ideology, systematic barriers, and unequal power relationship influence women's and marginalized farmers' equitable access to resources, opportunities, and space for meaningful participation in the irrigation and agriculture sector [29,40,41]. With the rise in male migration, the significance of women, elderly individuals, and marginalized farmers becomes crucial in managing water resources, agricultural production, and ensuring food security. However, several factors perpetuate inequality in accessing and utilizing irrigation services and benefits for these groups of farmers. Sociocultural barriers, including caste and ethnicity-based discrimination, further restrict access to resources, opportunities, and participation [42]. Women's competencies in irrigation and leadership face scrutiny, while institutional barriers and inadequate support hinder progress. Hence, it is essential to address gender, social inclusion, and governance-related factors in the irrigation and agriculture sector to achieve equitable and sustainable outcomes.

While there are studies that provide overviews of irrigation and agricultural management challenges, they are focused on themes such as physical infrastructure [43], agriculture water management, socio-economic factors and market access [27,28] or policy, governance and gender and social inclusion (GESI) [43,44]. Interdisciplinary studies that compare across and at the intersection of the various themes to assess trade-offs are limited due to disciplinary-focused studies. Characterizing challenges at the system level is of utmost importance as it facilitates the implementation of targeted measures to address the problem and improve water productivity. It also allows for the provision of tailored advisory services specifically designed to address these challenges, thereby contributing to an overall improvement in food productivity. In Nepal, deriving lessons from various parts of the system can create possibilities for replicating promising practices in other systems with similar geographical and socioeconomic conditions.

To understand impediments to effective irrigation water management in Nepal, this paper assessed and ranked the challenges of irrigation water management in three irrigation systems in Western Nepal namely Babai, Rani Jamara Kulariya and Mahakali. These schemes were selected as they reflect the three main schemes in Western Nepal. They are also considered increasingly representative of irrigation schemes in Nepal as they are large, managed by government, and face some challenges in achieving year-round cultivation. To evaluate challenges on the three schemes, we collected perceptions from 449 farmers using a Likert scale across 33 indicators highlighting key barriers to effective irrigation and agricultural management in four broad thematic areas namely: physical and structural, agricultural and water, socioeconomic and market, and gender and governance. This analysis enabled comparison of the relative importance of various challenges across the three irrigation schemes, different locations within the schemes (i.e. head, mid and tail sections of the system), and thematic areas.

## 2 Materials and methods

### 2.1 Study area

**Fig. 1:**
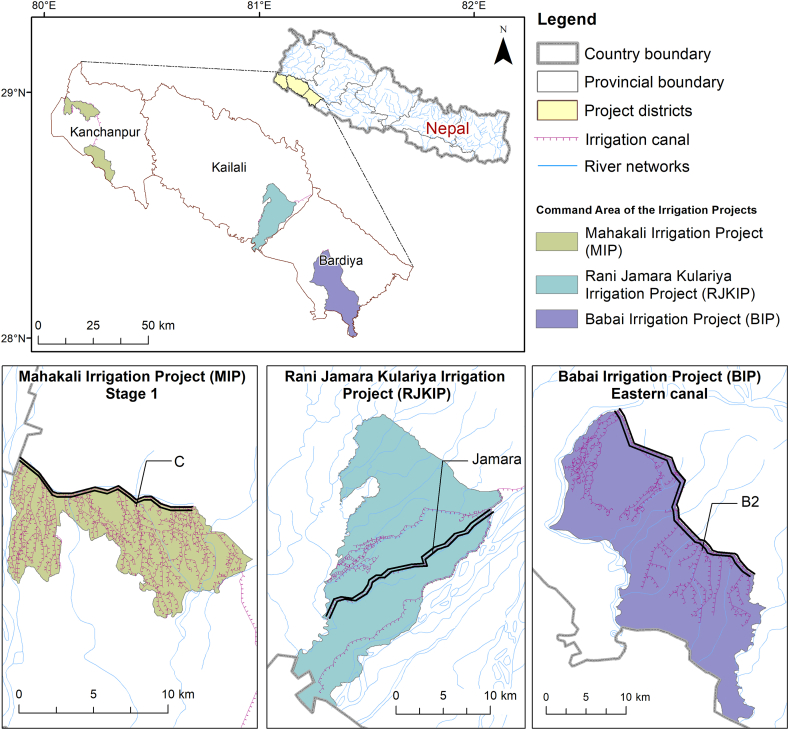
Map of the three irrigation systems (from west to east): Mahakali Irrigation Project (MIP), Rani Jamara Kulariya Irrigation Project (RJKIP), Babai Irrigation Project (BIP). The main canal in each systems is highlighted using parallel black lines. The surveyed canal for each scheme is specifically labelled. C in MIP, Jamara in RJKIP and B2 in BIP are the canal systems which were chosen for the respondent surveys.

Three irrigation schemes from western Nepal were examined, namely from west to east: the Mahakali Irrigation Project (**MIP**), Rani Jamara Kulariya Irrigation Project (**RJKIP**), and Babai Irrigation Project (**BIP**) (Fig. 1, Table 1). They are gravity-fed surface irrigation systems either supported by agency or farmer-managed systems. The average annual rainfall in Nepal is 1,390 mm and 80 % occurs during the monsoon period (June–September) which may result in insufficient water availability during other seasons.

**Table 1:** Salient features of three irrigation systems.

Characteristics	Mahakali Irrigation Project	Rani Jamara Kulariya Irrigation Project	Babai Irrigation Project
**Design discharge (Main Canal)** **(Cumecs)**	28.35	55	57 Eastern canal: 27.48 Western canal: 30
**Minimum flow in the river (dry season flow)** **(Cumecs)**	130	300	5
**Command area** **Irrigated land**	45,120 ha 5100 ha (stage I: 2028–2044 BS) 6500 ha (stage II: 2046–2054 BS) 33520 ha (stage III)	38,300 ha (including extension beyond RJK system using the same main canal)	36,000 ha Eastern canal: 21,000 ha Western canal: 15,000 ha
**Sources of Water**	Mahakali River	Karnali River	Babai River
**Catchment area (km**^**2**^**)**	15,260	44,000	2966
**Major crops**	Paddy: Monsoon Wheat: Winter Maize: Spring	Paddy: Monsoon Wheat: Winter Maize: Spring	Paddy: Monsoon Wheat: Winter Mustard: Winter
**Location (District)**	Kanchanpur (7 Municipalities and 1 Rural Municipality)	Kailali (8 Rural Municipalities and 1 Municipality (Tikapur))	Bardiya (5 Municipalities and 1 Rural Municipality)
**Institutions**	• WUAs for the Mahakali irrigation project •Sub WUAs for respective canals; •Maintenance and operation by the government-led Mahakali irrigation office	•WUAs for the Rani irrigation system •WUAs for the Jamara irrigation system •WUAs for the Kulariya irrigation system •Maintenance and operation by the government-led Rani-Jamara-Kulariya irrigation office	•WUAs for the Babai irrigation scheme •Sub WUAs for respective canals; •Maintenance and operation by the government-led Babai irrigation office

All three schemes receive government support. MIP and BIP are supported solely by the government of Nepal, whereas RJKIP receives government and 10.13039/100004421World Bank support. All these irrigation systems in western Nepal have distinct characteristics and are considered "National Pride Projects" by the government, and face both common and unique challenges in irrigation and agricultural management. In all irrigation systems, there are WUAs with interconnected responsibilities. At the highest level, there is a main committee that includes adequate representation from the branch levels. Likewise, each branch canal system has separate branch committees of WUA. The government-led irrigation offices work closely with the WUAs to ensure the proper maintenance and operation of these systems. The responsibility of operation and maintenance is shared between the government and the WUA in accordance with the canal hierarchy. These projects are currently in varying stages of development. BIP is still working on expanding its irrigation infrastructure towards the tail section whereas RJKIP is in the stage of command area development and soon preparing to transfer responsibilities to WUAs. MIP has completed the construction phase and has even commenced irrigation service fee collection, and the project scope is largely limited to small-scale repair and maintenance work. Given their distinct phases of development, each project presents its unique set of challenges and opportunities.

More detail on each scheme is provided here.

**MIP** is a transboundary irrigation scheme along the Nepalese border with India. MIP is a newly planned and constructed system by the government. As per the 1996 agreement between India and Nepal for the Integrated Development of Mahakali Barrage including Sarada Barrage, Tanakpur Barrage and Pancheswar Project, Nepal is entitled to receive a supply of 28.35 cumecs of water during the wet season and 4.25 cumecs during the dry season from the Mahakali River [45]. The project diverts water from the river at the Sarada Barrage into Nepal via the conveyance canal. MIP is being developed in three stages. Stage I and II are completed and providing irrigation to 11,600 ha utilizing water from the Sarada Barrage. Stage III is ongoing and intends to provide irrigation to 33,520 ha with water supplied from the Tanakpur Barrage.

**RJKIP** is one of Nepal's largest and oldest farmer managed irrigation systems, with only a minor expansion of the command area following government intervention. RJKIP is among the most prominent Farmer Managed Irrigation Systems (FMIS) in the Terai with a total cultivable command area of 14,300 ha, of which about 11,000 is currently irrigated. Initially, these irrigation systems had temporary headworks in the Karnali River and were traditionally operated and managed by the indigenous Tharu community. Later, towards the end of 1986, all these three systems were integrated into the Rani, Jamara, and Kulariya Irrigation System [46]. An integrated headwork along with the canal system at Chisapani started in 2023 delivering irrigation water (although the study survey was conducted before this and considered as an individual scheme). The RJKIP aims to modernise the irrigation infrastructure, improve water productivity, and strengthen farmer organizations and biodiversity conservation in the RJK command areas.

**BIP** is a key irrigation scheme along the Babai river, a tributary to the Karnali. BIP is a hybrid system, with farmers managing the head reaches and government funding used to irrigate a large part of the command area after the construction of a diversion weir and associated canal infrastructure. BIP aims to irrigate 36,000 ha, of which 21,000 ha is on the eastern side, and 15,000 ha on the western side of the Babai River, but currently, only 25,000 ha (69 %) has irrigation infrastructure. In the eastern canal area, only 81 % of the land has irrigation facilities during summer and 57 % in winter. Only 53 % of the land area in the western canal is irrigated so far. Continuous effort is needed to expand the irrigation canals to achieve the maximum command areas. The average maximum discharge of the Babai River is approximately 7,490 cumecs. In dry seasons (mid-April to mid-July) the discharge decreases to 5 cumecs. An inter-basin water transfer project is underway via the Bheri Babai diversion multi-purpose project (BBDMP). This project aims to bring 40 cumecs of water from the Bheri River to the Babai River through a 12.2 km tunnel. As a result, the BIP will provide consistent year-round irrigation to 51,000 ha of agricultural land in the Banke and Bardiya districts.

### 2.2 Methods and approach

#### 2.2.1 Identifying irrigation and agriculture performance indicators

To identify irrigation and agriculture performance indicators, we first selected indicators that reveal management challenges based on literature focused on holistic irrigation and agricultural management [[13], [14], [15],^29^,^47^,^48^]. The broad themes that emerged were structural, agriculture, gender, governance, socio-economic and markets. In addition, two field visits were carried out in August and October 2022 in the three irrigation systems to consult with various stakeholders, particularly the WUAs and farmers who provided contextual challenges of these irrigation systems. First, we categorized these challenges into four broad thematic categories just mentioned, namely **physical and structural** (9 indicators), **agriculture and water** (10), **socioeconomic and market** (7), and **GESI and governance** (7). Ultimately, 33 indicators were utilized to contextualize the irrigation and agriculture management challenges (Table 2).

**Table 2:** Selected irrigation performance indicators.^1^.

Categories	Indicators	Definitions	Sources
Physical and structural	Sedimentation in Monsoon	Sedimentation and siltation problems in canals during the monsoon season	Paudel [20]
	Sedimentation in Winter	Sedimentation and siltation in winter	Paudel [20]
	Insufficiency of water at fields	Problem of irrigation water not reaching the field	Stakeholder consultation
	Leakages from canals	Problem of water leaking in the irrigation canal	Stakeholder consultation
	Water logging	Problem of water logging around irrigation canals	Stakeholder consultation
	Mud and sedimentation problem	Problem of muddy soil, sand, and sedimentation	Paudel [20]
	Canal destroyed by flood	Problem of irrigation canals destroyed by flooding	Adhikari [18] and Howarth & Lal [19]
	Maintenance of irrigation system	Problems with the maintenance of irrigation systems	Stakeholder consultation
	Maintenance of irrigation canal	Level of challenges of not doing regular maintenance of irrigation canal	Stakeholder consultation
Agricultural and water management	Winter water availability	Problems of getting enough water in winter	Dhakal et al. [49] and field visit
	Monsoon water availability	Problems of getting enough water in monsoon	Dhakal et al. [49] and field visit
	Spring water availability	Problems of getting enough water in Spring	Dhakal et al. [49] and field visit
	Agricultural advice on time	Problem of getting agricultural advice from government office on time	Gajmer [23] and field visit
	Operating agricultural equipment	Problem of operating agricultural equipment	DWRI [3] Field Visit
	Intercultural operations due to lack of water	Problem faced in spraying fertilizer, tilling planting seeds, etc, due to lack of water	Stakeholder consultation
	Use of alternative irrigation method	Problems in accessing alternative methods of irrigation (such as drip and sprinklers)	Stakeholder consultation
	Lack of knowledge on A&I	Problems faced by the farmers due to lack of knowledge or information regarding agriculture and irrigation	Stakeholder consultation
	Soil fertility problem-Monsoon	Problem of soil fertility in monsoon	Stakeholder consultation
	Soil fertility problem-Winter	Problem of soil fertility in winter	Stakeholder consultation
GESI and governance	Contacting irrigation officials	Difficulty in contacting government irrigation officials	Stakeholder consultation
	Contacting agricultural officials	Difficulty in contacting government agricultural officials	Stakeholder consultation
	Availability of agri-mechanization supports	Problems in availing various facilities (subsidies, loans, etc.) for agricultural mechanization	Field visit; Paudel et al. [50]
	Agricultural subsidies from agencies	Problem faced in getting agricultural subsidies from relevant agencies	Stakeholder consultation
	Technical support for agriculture and irrigation (A&I)	Problem of getting technical assistance related to agriculture and irrigation	Stakeholder consultation
	Information related to A&I	Problem of getting information related to agriculture and irrigation?	Stakeholder consultation
	Participation in WUA activities	Problem in actively participating in discussion in WUA due to social and cultural reasons	Khadka et al. [29] and ADB [42]
Socio-economic and markets	Labour availability	Problems of agricultural labour during the farming season	Stakeholder consultation
	Access to capital	Problem due to a lack of capital for irrigation and agriculture work	Clement et al. [35]; Tadesse [36]
	Access to fertilizer	Problem of timely availability and adequacy of fertilizer	[29,37]
	Access to quality seed	Problem of availability and adequacy of quality seeds	[51]
	Fair market price	Problem of not getting a good market price	Krupnik et al. [27,28]
	Access to output markets	Problem of a market for selling agricultural produce	Krupnik et al. [27,28]
	Dispute between WUA members	Problem of a dispute between WUA members/committee member	Stakeholder consultation

#### 2.2.2 Irrigation challenges score

Using the 33 irrigation performance indicators, we generated the **Irrigation Challenges Score** (Equation (1)). A five-item Likert scale i.e., **very high (5), high (4), moderate (3), low (2) and very low (1)** was utilized to support the application of each indicator. Respondents from three irrigation projects provided their perceptions on each indicator using the scale [[52], [53], [54]]. A consolidated challenge score for each indicator was developed based on the weighted average where the number of respondents (Table 3) is taken as the weights. These weights are taken at each step of calculating the challenge score for each scheme (i.e. head, mid and tail section of each irrigation system).

Equation 1 $$Challenge\;Score=\frac{\sum_{i=1}^{i=5}W_{i}X_{i}}{\sum_{i=1}^{i=5}W_{i}}$$

**Table 3:** Total households and sampled households in Head, Mid and Tail regions of the selected project in the study area.

Irrigation Project	Total HHs of the system	Total HHs in the selected canals (N)			Sampled HHs (n) (% of total N population)			Total Sampled HHs
		Head	Mid	Tail	Head	Mid	Tail	
**MIP (Stage I & II)**	20,897	400	1,200	450	50 (13 %)	47 (4 %)	48 (11 %)	145
**RJKIP**	34,589	800	1,000	900	61 (8 %)	49 (5 %)	50 (6 %)	160
**BIP (East & West)**	59,216	750	1,000	700	48 (6 %)	48 (5 %)	48 (7 %)	144
Total	114,702	1,950	3,200	2050	159 (8 %)	144 (5 %)	146 (7 %)	449

Note: The number of HHs in each sample area is approximate and the information was received from the representatives of WUAs of each canal due to a lack of exact HH data.

Where X_i_ is the numerical ranking score for each indicator and W_i_ is the number of positive responses for the given score.

The range of challenge scores is from 1 to 5 where 1 represents a very low challenge and 5 represents a very high challenge. The aggregated challenge score was developed for each section (head, mid and tail) of the irrigation systems, each irrigation scheme and for thematic areas.

#### 2.2.3 Sampling and data collection

Canal selection.

**Fig. 2:**
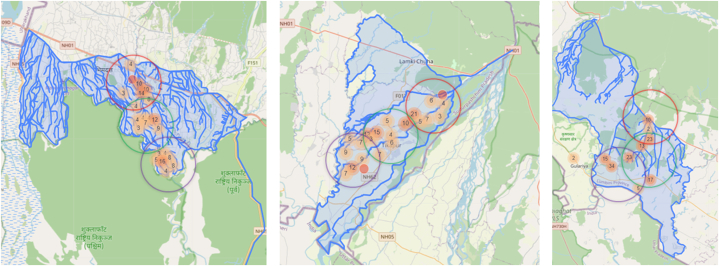
Location of the sample survey HHs in the select canals of three irrigation systems. The circle indicates the tentative demarcation of the head (red), mid (green) and tail (purple) sections of the selected canals for the survey. Note: The image was obtained from the Kobo platform, which was used to collect and store sample household data.The data was then overlaid on GIS maps of irrigation canals. The numbers inside the bubbles indicate the number of households surveyed in that area. Circles outside the boundary of the irrigation systems indicate that the respondents' houses, where the interviews took place, were outside, but their fields were inside the command area.

The sample data selection was done in two stages. In the first stage, we selected a branch canal from the main canals of each scheme for data collection. We purposively selected a canal from each system that represented the characteristics and features of the other canals within the systems, as they were positioned within the middle of the main canal system. Within a canal, heterogeneity exists among farmers in water availability and access resulting in differential water and agricultural productivity. To enhance analysis insights, we adopted Şener et al. [55] and Agide et al. [56] who proposed dividing the canal into head, mid, and tail reaches (Fig. 2a, b, 2c). In this study, we stratified selected canals from three systems based on their physical proximity to the main canal (i.e., source) categorising them into head, mid and tail farmers. Within the selected canal system, head, mid and tail irrigators were delineated on an approximately equal distance basis considering the total length of the selected canal.

BIP has two main canal systems: the Eastern Canal and the Western Canal. For our study, we selected the Eastern Canal of BIP as this canal is more operational compared to the Western Canal. The Eastern Canal has 23 branch canals (four main canals and 19 subsidiary canals). We selected the B2 branch canal located in the middle part of the main canal that has a canal length of about 15 km with a 4,850 ha command area. Once the branch canal is selected, the canal is further divided into three areas (head, mid and tail) across the length of the branch canal. Table 3 provides detailed information about the total and sampled HHs of the head, mid and tail regions of the selected canals.

In RJKIP, there are three canal systems namely Rani, Jamara, and Kulariya, which originated from 3 different intakes from the Karnali River and were earlier considered as a separate system. Out of three canal systems, we selected Jamara, which lies in between the Rani and Kulariya. 11 subsidiary branches originate from the Jamara main canal as listed below. These subsidiary canals are divided into three sections (Section 1, Section 2, Section 3). Section 1 is treated as head, Section 2 as mid and Section 3 as tail across the length of the canal. An integrated intake system has operated and provides irrigation water to all canal systems since 2023.

In MIP, we selected MIP-1 (stage 1) as this is the complete irrigation system (MIP stage 2 is still under expansion). Five main branches originate from the MIP-1 named Canals A, B, C, D and E. Out of five canals, the C canal was selected as this lies in the middle section of the main canal and is divided into three sections i.e., head, mid and tail.

Survey method.

The household survey was carried out within four months from November 2022 to February 2023 by deploying local enumerators who were trained before the data collection in the Kobo platform using mobile devices. The identification of households located in head, mid, and tail regions in each survey location (which was selected in the first stage) was a crucial step in the process. The households were identified through field observation (transect walk) followed by consultation with the representative of the WUA in each region (head, mid and tail). The number of households in each region was determined after consulting with the WUA representatives (Table 3).^2^ The samples from each survey area were collected using a ‘random walk’ protocol [57,58] in the absence of a detailed household roster. Following the ‘random walk’ protocol, a landmark (which lies at the centre) in each survey area (head, mid and tail) was chosen to serve as the starting point for the data collection. A household was selected nearest to the landmark in each survey area for data collection. The following households were selected by identifying every fourth household along the canal. If the household identified was not involved in the agricultural activities within the survey area or family members were not present during the time of the survey, the household next to the 4th household was selected for data collection. A total of 449 samples were collected for the study.

The sample in each cluster (head, mid, tail) was fixed using Cochran's method suggested by Singh & Masuku [59]. Table 3 shows the number of households in the three systems, the distribution of households in the selected canals i.e., head, mid and tail and then the number of sampled households from each stratum.

This study was approved by the Institutional Review Board of the International Water Management Institute (IWMI) with ethics approval reference #2022_24. Also, informed consent was obtained from all participants during the survey, focus group discussions and validation meetings.

Validation meetings.

After the field survey, validation meetings were conducted from the 26th to the July 29, 2023. Meetings were held in each canal system where the survey was carried out. The validation meetings were aimed at confirming findings and clarifying reasons explaining challenges identified for the particular canal system.

Limitations.

The research was conducted in specific canals within the irrigation systems of BIP and MIP. In the case of RJKIP, only the Jamara irrigation system was included. The selected canals were chosen from the middle of the main canals to ensure that the responses from these canal systems are representative of the entire system. Due to the absence of a list of the entire population, the 'random walk' protocol for household selection was adopted, which may potentially lead to sampling bias. Additionally, the surveyed population ranged from 5 % to 13 % of the total population of the respective canals. The survey took place between November and February 2023 (off time of irrigation management) and the response was collected on a recall basis and there may be bias in response due to the timing of the survey.

## 3 Results

### 3.1 Irrigation and cropping intensity

The major cropping system is cereal-based with monsoon rice and winter wheat. Winter mustard and potato are cultivated in some areas. Summer maize is also practised in rainfed conditions in a few areas. Interestingly most farmers were not cultivating spring paddy or maize, though there is scope to add either one of these crops in the cropping sequence. Based on the respondent survey, the average landholding size was 0.9 ha in the three irrigation systems. The landholding distribution among the three systems ranged from 0.52 to 1.4 ha (Table 4) [60]. The cropping calendar in all three systems was almost the same and the average cropping intensity (CI) was 289 % for all systems (minimum 274 % for MIP-Tail and maximum 299 % for Head in MIP). The National Water Plan (NWP) 2005 target for CI is 193 % in 2027. While the [81] aims for a CI of 230 % by 2045. Given the national standard, the CI in all three systems exceeds national targets. The average cropping intensity was higher than the national average of 163 % in 2019 [10]. This is because all the land within these schemes has an irrigation command area, which naturally results in higher cropping intensity than the national figure, including the rain-fed system.

**Table 4:** Cropped area and cropping intensity in the command area (2021-22).

Project	Command area	Average holding size (ha)	Cropping intensity (%)	Rice yield (ton/ha)	Wheat yield (ton/ha)
MIP (145)	Head (n = 50)	0.65 (0.92)	299	2.6 (40)	1.0 (38)
	Mid (n = 47)	0.51 (0.32)	297	3.0 (45)	1.3 (44)
	Tail (n = 48)	0.67 (0.49)	274	3.5 (44)	2.1(37)
RJKIP (160)	Head (n = 61)	1.03 (0.93)	290	4.4 (52)	3.9 (18)
	Mid (n = 49)	1.41 (1.06)	291	3.6 (49)	2.8 (44)
	Tail (50)	1.28 (1.59)	279	3.6 (30)	3.9 (20)
BIP (144)	Head (n = 48)	0.52 (0.43)	297	3.3 (48)	1.5 (47)
	Mid (n = 48)	1.00 (0.86)	298	4.0 (37)	1.8 (34)
	Tail (n = 48)	0.87 (0.77)	278	3.7 (48)	2.9(42)
Total (n = 449)		**0.90 (0.95)**	**289**	3.6 (393)	2.1(324)
Lumbini province				3.9	3.4
Sudhurpaschim province				3.6	2.5
National				3.8	2.9

The average yield of rice and wheat achieved in the three irrigation systems is presented in Table 4. These yields are comparable with provincial and national averages considering rain-fed yield and irrigated yields. However, notable yield differences exist, such as MIP's head which generates a wheat yield of only 1 ton/ha which is one-third of the national average and less than half that of the province.

It was found that farmers can get higher yields when the sowing time is appropriate, and irrigation is available during the critical phases of the crop development. However, wheat planting time is delayed or if farmers fail to irrigate their wheat crop three weeks after sowing, yield reduces drastically. In our discussion, some farmers pointed out that farmers from the head region did not get water in the canal in winter due to repair and maintenance work while the tail region farmers irrigated their crops using groundwater. As per the farmers, the wheat flowering period was impacted by a westerly wind in 2021, resulting in flower damage and a decrease in the wheat yield. Upadhyaya [61] also found a significant reduction in wheat yield due to westerly wind in the western part of Nepal.

### 3.2 Challenges in the irrigation projects

#### 3.2.1 Physical and structural

The three highest-ranked challenges for physical and structural are i) Insufficiency of water at fields (average challenge score: 3.6), ii) maintenance of irrigation canals (3.5), and iii) leakages from canals (3.4) (Table 5). In most cases, the highest-ranked challenge within each scheme was also within the top three ranked average challenges. Conversely, the three smallest challenges were sedimentation in monsoon, mud and sedimentation problem, and sedimentation in winter (Table 5). Based on the average challenge score, BIP seems to have the greatest challenges (3.0) followed by RJKIP (2.8).

**Table 5:** Challenge scores of the indicators of the physical and structural challenges. The average values are taken from scores from three irrigation systems and ranking is based on the average rank of the three systems.

Indicators	Challenge Scores				Average Rank
	MIP	RJKIP	BIP	Average	
**Insufficiency of water at fields**	3.3	3.6	4.0	3.6	**1**
**Maintenance of irrigation canal**	3.5	3.2	3.9	3.5	**2**
**Leakages from canals**	3.4	3.0	3.8	3.4	**3**
Maintenance of irrigation system	3.2	3.3	3.6	3.4	4
Water logging	2.8	2.5	3.4	2.9	5
Canal destroyed by flood	1.9	3.1	2.9	2.6	6
Sedimentation in Monsoon	2.0	2.4	2.0	2.2	7
Mud and sedimentation problem	1.7	2.2	1.8	1.9	8
Sedimentation in Winter	1.6	2.1	2.0	1.9	9
Average	**2.6**	**2.8**	**3.0**		

Note: MIP: Mahakali Irrigation Project; RJKIP: Rani Jamara Kulariya Irrigation Project; and BIP: Babai Irrigation Project.

#### 3.2.2 Agricultural water management

In agricultural water management, the three highest-ranked challenges are i) spring water availability (average challenge score: 4.3), ii) intercultural operations due to lack of water (4.0), and iii) winter water availability (3.8) (Table 6). In most cases, the highest-ranked challenge within each scheme was the same as the top-ranked average challenge. Conversely, the three smallest challenges were operating agricultural equipment, soil fertility problem in winter, and soil fertility problem in monsoon. Based on the average challenge score, BIP seems to face the greatest challenges (3.8) closely followed by RJKIP (3.7).

**Table 6:** Challenge score of the agricultural water management in all 3 Irrigation Projects (IPs).

Indicators	Challenge Scores				Average Rank
	MIP	RJKIP	BIP	Average	
**Spring water availability**	3.8	4.6	4.3	4.3	**1**
**Intercultural operations due to lack of water**	3.5	4.3	4.3	4.0	**2**
**Winter water availability**	3.0	4.4	3.9	3.8	**3**
Lack of knowledge on A&I	3.4	3.8	4.1	3.8	4
Agricultural advice on time	3.4	3.9	3.9	3.7	5
Use of alternative irrigation method	3.3	3.6	3.9	3.6	6
Monsoon water availability	3.3	3.5	3.9	3.6	7
Operating agricultural equipment	2.8	3.7	4.0	3.5	8
Soil fertility problem-Winter	2.9	2.5	2.9	2.8	9
Soil fertility problem-Monsoon	2.8	2.4	2.7	2.6	10
Average	**3.2**	**3.7**	**3.8**		

Note: MIP: Mahakali Irrigation Project; RJKIP: Rani Jamara Kulariya Irrigation Project; and BIP: Babai Irrigation Project; A & I: Agriculture and irrigation.

#### 3.2.3 Socioeconomic and market challenges

For socio-economic and markets, the three highest-ranked challenges are i) access to fertilizer (average challenge score: (4.4), ii) fair market price (4.2), and iii) access to input-output market (4.2) (Table 7). However, the ranking differs from the average score within an individual scheme. For example, fair market prices is the top-ranked challenge in RJKIP whereas fair market price and access to input-output market are the first and second-ranked challenges (with the same score). Conversely, the three least smallest challenges were access to capital, labour availability and disputes between WUA members. Based on the average challenge score, BIP faces the biggest challenges (4.1) followed by RJKIP (4.0).

**Table 7:** Challenge score for the socio-economic challenge in all 3 Irrigation Projects (IPs).

Indicators	Challenge Scores				Average Rank
	MIP	RJKIP	BIP	Average	
**Access to fertilizer**	4.1	4.6	4.5	4.4	**1**
**Fair market price**	3.2	4.8	4.7	4.2	**2**
**Access to input-output market**	3.2	4.6	4.7	4.2	**3**
Access to quality seed	3.7	4.2	4.4	4.1	4
Access to capital	3.5	3.8	4.4	3.9	5
Labour availability	2.6	3.4	3.2	3.1	6
Disputes between WUA members	2.5	2.9	3.1	2.8	7
Average	**3.3**	**4.0**	**4.1**		

Note: MIP: Mahakali Irrigation Project; RJKIP: Rani Jamara Kulariya Irrigation Project; and BIP: Babai Irrigation Project.

#### 3.2.4 GESI and governance

For GESI and governance, the three highest-ranked challenges are i) agricultural subsidies from agencies (average challenge score: Male: 4.1 and Female 4.0), ii) contacting agricultural officials (male/female: 3.9) and iii) Agricultural mechanization support (male/female: 3.9) (Table 8). Conversely, the three least challenging indicators are Information related to A&I, Technical support for A&I, and Participation in WUA activities. For some challenges, the average score for males and females differs. Notably, in RJKIP, for each indicator, the challenge score given by females is higher than males (average: male 3.7 and female: 3.9). In MIP, the male score is higher than the female score, except for participation in WUA activities. In BIP, the challenge score is mixed for both males and females. For one indicator ‘participation in WUA activities’, in all irrigation systems, female participants gave higher scores than male participants indicating more challenge in the participation of the females. Similarly, in RJKIP, the largest differences in scores of males and females are in contacting irrigation officials (average rank #4) (male: 3.6 and female 4.0) whereas in MIP, this is with agricultural subsidies from agencies (rank #1) (male 3.7 and female 3.4) and with BIP, this is with three indicators ranked as #2, 4 and 7).

**Table 8:** Percentage distribution of the GESI and Governance challenges indicators in all 3 Irrigation Projects (IPs).

Indicators	MIP		RJKIP		BIP		Average		Over all score	Rank M	Rank F	Aver age rank
	M	F	M	F	M	F	M	F				
**Agricultural subsidies from agencies**	**3.7**	**3.4**	4.2	4.3	4.5	4.4	4.1	4.0	4.1	1	1	1
**Contacting agricultural officials**	4.1	3.9	3.7	4.0	**3.9**	**3.7**	3.9	3.9	3.9	2	3	2
**Agricultural mechanization support**	3.9	3.6	3.9	4.1	3.8	3.9	3.9	3.9	3.9	3	2	3
Contacting irrigation officials	3.9	3.8	**3.6**	**4.0**	**3.9**	**3.7**	3.8	3.8	3.8	5	4	4
Information related to A&I	3.7	3.5	3.6	3.9	4.1	4.1	3.8	3.8	3.8	4	5	5
Technical support for A&I	3.6	3.5	3.6	3.8	4.1	4.1	3.8	3.8	3.8	6	6	6
Participation in WUA activities	2.5	2.7	3.0	3.3	**3.5**	**3.7**	3.0	3.2	3.1	7	7	7
Average	**3.6**	**3.5**	**3.7**	**3.9**	**4.0**	**4.0**	**3.8**	**3.8**				

Note: MIP: Mahakali Irrigation Project; RJKIP: Rani Jamara Kulariya Irrigation Project; and BIP: Babai Irrigation Project; A & I: Agriculture and irrigation; WUA: Water users associations. M: Male and F: Female.

### 3.3 Comparison of challenges across the three system

**Fig. 3:**
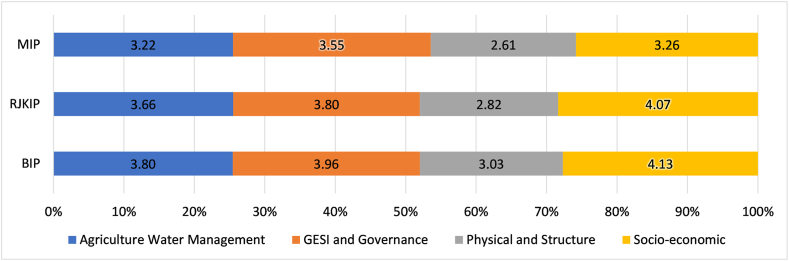
Category-wise distribution of total challenge scores in all three irrigation projects. The data label shows the average challenge score for each category. The highest score for each project is highlighted.

Fig. 3 compares challenges in four categories across the three irrigation systems. The socio-economic challenges, on average, were indicated as the most critical challenges in all irrigation systems. This alone accounts for 27.4 % of the challenges closely followed by GESI and governance (26.9 %), agricultural water management (25.5 %) and physical and structural (20.2 %). While socio-economic challenges have the highest score for RJKIP and BIP, GESI and Governance issues take the forefront in MIP.

Comparing the average challenge score for all segments of the three projects (Table 9), the highest score is seen in BIP–Mid (Challenge score: 4.5), followed by RJKIP–Tail (3.7) and RJKIP–Mid (3.6). The lowest problem is reported in MIP–Head (2.8), followed by BIP–Head (3.2).

**Table 9:** Average challenge score for irrigation regions.

Irrigation region	Challenge score	Ranking
**BIP–Mid**	4.5	1
**RJKIP–Tail**	3.7	2
**RJKIP–Mid**	3.6	3
**MIP–Tail**	3.5	4
**BIP–Tail**	3.5	5
**RJKIP–Head**	3.5	6
**MIP–Mid**	3.2	7
**BIP–Head**	3.2	8
**MIP–Head**	2.8	9

Comparing the average challenge score for the head, mid and tail of the three projects for four categories (Table 10), the highest score is associated with the mid region of the irrigation systems(Challenge score: 3.79), followed by Tail (3.56).

**Table 10:** Category-wise distribution of total challenge scores in all 3 Irrigation Projects.

Category	Head	Mid	Tail	Average
**Agriculture Water Management**	3.21	3.75	3.76	**3.56**
**GESI and Governance**	3.32	**4.04**	**4.00**	**3.77**
**Physical and Structural**	2.56	3.29	2.63	**2.82**
**Socio-economic and market**	**3.57**	4.09	3.84	**3.83**
Average	**3.17**	**3.79**	**3.56**	

Table 11 shows the consolidated challenge score for each section of the irrigation systems, the average score for the systems and the overall average score. ‘Water availability in the spring season’ is the top-ranked challenge within the agricultural water management category for six out of nine (6/9) sections of the three irrigation systems followed by ‘Intercultural operations due to lack of water’(2/9) and ‘Lack of knowledge on agriculture and irrigation’ (1/9). ‘Agricultural subsidies from agencies’ is the top-ranked challenge within the GESI and governance category with three out of nine sections of the three irrigation systems followed closely by ‘Information related to agriculture and irrigation’(2/9), ‘Contacting irrigation officials’ (2/9), and ‘Availability of agri-mechanization supports’ (2/9). ‘Sufficiency of water at fields’ is the top-ranked challenge within the physical and structure category for all but four sections of the three irrigation systems closely followed by ‘Maintenance of irrigation canal’(3/9). ‘Maintenance of irrigation system’ is an issue in only one section (BIP–Mid). ‘Access to fertilizer’ (4/9) and ‘Fair market price’ (3/9) are the top two ranked challenges within the socio-economic and market category within the nine sections of the three irrigation systems. ‘Access to output markets’ (1/9) and ‘Access to capital’ (1/9) are the top issues in BIP–head and BIP–Mid respectively.

**Table 11:** Combined and individual challenge scores of 3 irrigation projects. The highlighted ranks are the top three challenges in the corresponding irrigation scheme. The challenge indicator in bold is the top-ranking issue within the categories. The standard deviation of the response is presented in the parenthesis).

Category	Challenge Indicators	MIP					RJKIP					BIP					Total	
		Head	Mid	Tail	Aver age	Rank	Head	Mid	Tail	Aver age	Rank	Head	Mid	Tail	Average	Rank	Overall average	Rank
Agriculture Water Management	Winter water availability	2.9 (0.94)	3.02 (0.93)	3.23 (1.05)	3.05 (0.98)	22	4.41 (0.71)	4.02 (0.51)	4.78 (0.46)	4.41 (0.58)	5	3.13 (0.33)	4.85 (0.35)	3.83 (0.96)	3.94 (0.62)	14	3.82 (0.74)	12
	Monsoon water availability	2.98 (1.03)	2.83 (1)	4.02 (0.97)	3.28 (1)	17	3.1 (1.18)	3.51 (0.54)	4.1 (0.73)	3.54 (0.89)	20	2.77 (0.42)	4.67 (0.66)	4.31 (0.92)	3.92 (0.69)	15	3.57 (0.87)	19
	Spring water availability	2.78 (0.99)	3.81 (1.2)	5.0 (0)	3.85 (0.89)	4	4.61 (0.84)	4.18 (0.44)	4.96 (0.2)	4.59 (0.58)	4	3.46 (0.5)	4.88 (0.33)	4.71 (0.5)	4.35 (0.45)	7	4.27 (0.67)	2
	Agricultural advice on time	2.7 (0.88)	3.4 (0.76)	4.15 (0.65)	3.41 (0.77)	14	3.82 (0.97)	3.67 (0.62)	4.12 (0.74)	3.87 (0.8)	10	2.73 (0.7)	4.83 (0.42)	4 (0.58)	3.85 (0.58)	19	3.71 (0.73)	16
	Operating agricultural equipment	2.82 (0.79)	2.79 (0.87)	2.88 (1.17)	2.83 (0.96)	24	3.74 (0.83)	3.65 (0.69)	3.6 (0.75)	3.67 (0.76)	17	3.6 (0.49)	4.83 (0.37)	3.46 (0.64)	3.97 (0.51)	12	3.49 (0.77)	21
	Intercultural operations due to lack of water	3.06 (0.93)	3.3 (0.85)	4.27 (0.7)	3.54 (0.83)	10	4.13 (0.88)	3.69 (0.79)	4.96 (0.2)	4.26 (0.7)	6	3.48 (0.58)	4.96 (0.2)	4.31 (0.58)	4.25 (0.49)	8	4.02 (0.69)	7
	Use of alternative irrigation method	2.66 (0.86)	3.4 (0.82)	3.77 (1.1)	3.27 (0.94)	18	3.7 (0.89)	3.73 (0.69)	3.3 (0.7)	3.59 (0.78)	19	3.58 (0.53)	4.9 (0.31)	3.17 (0.85)	3.88 (0.61)	17	3.58 (0.78)	18
	Lack of knowledge on A&I	2.84 (0.9)	3.45 (0.71)	3.79 (0.76)	3.35 (0.8)	15	3.74 (0.77)	3.41 (0.53)	4.16 (0.81)	3.77 (0.72)	15	3.83 (0.47)	4.9 (0.42)	3.71 (0.5)	4.15 (0.46)	9	3.76 (0.68)	15
	Soil fertility problem-Monsoon	2.2 (0.96)	2.68 (0.72)	3.46 (0.89)	2.77 (0.86)	26	2.33 (0.88)	2.9 (0.61)	2.12 (1.05)	2.44 (0.87)	30	2.83 (0.62)	3 (0.5)	2.33 (1.36)	2.72 (0.91)	30	2.64 (0.88)	29
	Soil fertility problem-Winter	2.24 (0.74)	3.02 (0.86)	3.42 (0.76)	2.88 (0.79)	23	2.46 (1.14)	2.96 (0.73)	2.04 (0.94)	2.48 (0.96)	29	2.96 (0.61)	3.08 (0.34)	2.75 (1.3)	2.93 (0.85)	28	2.76 (0.87)	28
GESI and Governance	Contacting irrigation officials	3.9 (0.41)	3.6 (0.89)	4.08 (0.53)	3.86 (0.64)	3	3.84 (1.03)	3.76 (0.48)	3.92 (1.18)	3.84 (0.95)	12	1.96 (0.54)	4.65 (0.52)	4.79 (0.5)	3.8 (0.52)	20	3.83 (0.74)	11
	Contacting agricultural officials	3.9 (0.61)	3.79 (0.71)	4.19 (0.53)	3.96 (0.62)	2	3.69 (0.98)	3.73 (0.56)	4.06 (1.08)	3.82 (0.91)	13	2 (0.68)	4.6 (0.57)	4.79 (0.5)	3.8 (0.59)	20	3.86 (0.73)	10
	Availability of agri-mechanization supports	3.18 (0.82)	3.81 (0.82)	4.31 (0.79)	3.76 (0.81)	5	3.89 (0.83)	4 (0.4)	4.16 (0.86)	4.01 (0.74)	9	3.92 (0.4)	4.92 (0.28)	2.85 (1.21)	3.9 (0.75)	16	3.89 (0.77)	9
	Agricultural subsidies from agencies	2.84 (0.92)	3.66 (0.81)	4.23 (0.65)	3.57 (0.8)	7	4.39 (0.71)	4.14 (0.53)	4.2 (0.85)	4.26 (0.71)	6	3.83 (0.42)	4.98 (0.14)	4.5 (0.5)	4.44 (0.39)	4	4.09 (0.66)	6
	Technical support for A&I	2.94 (0.86)	3.64 (0.63)	4.1 (0.55)	3.55 (0.7)	9	3.21 (0.91)	3.67 (0.55)	4.3 (0.88)	3.69 (0.8)	16	3.17 (0.66)	4.94 (0.24)	4.21 (0.61)	4.1 (0.54)	10	3.78 (0.69)	14
	Information related to A&I	2.66 (0.68)	3.68 (0.95)	4.4 (0.6)	3.57 (0.76)	7	3.43 (1)	3.61 (0.53)	4.38 (0.89)	3.78 (0.85)	14	3.17 (0.62)	4.98 (0.14)	4.1 (0.51)	4.08 (0.47)	11	3.81 (0.71)	13
	Participation in WUA activities	2.98 (0.65)	2.66 (0.88)	2.08 (0.57)	2.58 (0.71)	28	3.28 (0.85)	3.02 (0.8)	3.26 (1.15)	3.19 (0.94)	24	2.81 (0.6)	4.9 (0.37)	3.15 (1.04)	3.62 (0.73)	23	3.13 (0.8)	24
Physical and Structure	Sedimentation in Monsoon	1.88 (0.84)	2.21 (0.54)	1.94 (0.47)	2.01 (0.64)	30	2.46 (0.97)	2.94 (0.89)	1.9 (1.04)	2.43 (0.97)	31	1.94 (0.56)	2.85 (1.08)	1.25 (0.97)	2.01 (0.9)	31	2.16 (0.85)	31
	Sedimentation in Winter	1.82 (0.79)	1.81 (0.87)	1.1 (0.31)	1.58 (0.7)	33	2.44 (1.05)	2.06 (0.31)	1.68 (0.84)	2.09 (0.82)	33	1.98 (0.59)	2.69 (0.82)	1.25 (0.97)	1.97 (0.81)	32	1.89 (0.78)	33
	Insufficiency of water at fields	2.94 (1.26)	3.4 (1.12)	3.6 (1.27)	3.31 (1.22)	16	3.05 (1.4)	3.76 (0.74)	4.12 (1.01)	3.6 (1.11)	18	3.44 (0.57)	4.63 (0.73)	3.81 (1.2)	3.96 (0.88)	13	3.62 (1.08)	17
	Leakages from canals	2.7 (1.15)	3.79 (0.99)	3.81 (0.95)	3.42 (1.04)	13	2.25 (1.05)	3.49 (0.93)	3.32 (1.01)	2.96 (1)	26	3.69 (0.62)	4.75 (0.52)	2.94 (1.09)	3.79 (0.78)	22	3.38 (0.95)	22
	Water logging	2.52 (1.14)	3.17 (1)	2.67 (1.43)	2.78 (1.2)	25	2.25 (1.29)	2.59 (0.97)	2.68 (1.3)	2.49 (1.2)	28	2.67 (0.69)	4.25 (1.03)	3.33 (1.64)	3.42 (1.19)	25	2.88 (1.2)	26
	Mud and sedimentation problem	1.68 (0.73)	1.94 (0.63)	1.58 (0.53)	1.73 (0.64)	32	2.18 (1)	2.63 (0.9)	1.8 (1)	2.2 (0.97)	32	1.96 (0.5)	2.35 (0.99)	1.0 (0)	1.77 (0.64)	33	1.91 (0.77)	32
	Canal destroyed by flood	1.88 (0.91)	2.11 (0.72)	1.69 (0.96)	1.89 (0.87)	31	2.56 (1.18)	3.45 (0.95)	3.34 (1.38)	3.08 (1.18)	25	2.52 (0.68)	4.19 (0.97)	1.98 (1.38)	2.9 (1.05)	29	2.63 (1.05)	30
	Maintenance of irrigation system	2.66 (0.97)	3.53 (0.82)	3.5 (0.84)	3.22 (0.88)	19	2.56 (0.93)	3.59 (0.75)	3.9 (0.92)	3.29 (0.88)	22	3.73 (0.64)	4.81 (0.44)	2.13 (0.75)	3.56 (0.62)	24	3.35 (0.81)	23
	Maintenance of irrigation canal	2.8 (0.98)	3.85 (0.62)	3.98 (0.48)	3.53 (0.73)	11	2.84 (1.15)	3.1 (0.79)	3.82 (0.95)	3.23 (0.99)	23	4 (0.58)	4.75 (0.48)	2.88 (0.99)	3.88 (0.72)	18	3.53 (0.83)	20
Socio-economic and market	Labour availability	2.42 (0.78)	2.89 (0.72)	2.65 (1.09)	2.65 (0.88)	27	4.03 (0.99)	3.39 (0.69)	2.7 (0.83)	3.42 (0.86)	21	3.06 (0.47)	4 (1.08)	2.42 (0.89)	3.16 (0.85)	26	3.09 (0.86)	25
	Access to capital	2.92 (0.89)	3.47 (0.82)	4.1 (0.74)	3.49 (0.82)	12	4.15 (0.9)	4.02 (0.55)	3.32 (0.71)	3.85 (0.75)	11	4.04 (0.71)	4.98 (0.14)	4.15 (0.46)	4.39 (0.49)	6	3.91 (0.7)	8
	Access to fertilizer	3.16 (0.92)	4.38 (0.81)	4.9 (0.37)	4.13 (0.74)	1	4.31 (0.86)	4.96 (0.2)	4.68 (0.68)	4.63 (0.66)	3	4.04 (0.41)	4.88 (0.39)	4.46 (0.58)	4.46 (0.46)	3	4.41 (0.64)	1
	Access to quality seed	3.08 (0.82)	3.74 (0.56)	4.33 (0.59)	3.71 (0.67)	6	4.18 (0.93)	3.82 (0.63)	4.72 (0.57)	4.24 (0.74)	8	4.1 (0.47)	4.85 (0.5)	4.29 (0.61)	4.42 (0.53)	5	4.12 (0.66)	5
	Fair market price	2.58 (0.85)	3.38 (0.7)	3.6 (0.78)	3.18 (0.78)	20	4.7 (0.58)	4.8 (0.4)	4.92 (0.27)	4.8 (0.45)	1	4.1 (0.37)	4.98 (0.14)	5 (0)	4.69 (0.23)	1	4.24 (0.54)	3
	Access to output markets	2.82 (0.89)	3.04 (0.54)	3.65 (0.75)	3.17 (0.74)	21	4.57 (0.71)	4.45 (0.61)	4.92 (0.27)	4.64 (0.57)	2	4.13 (0.44)	4.98 (0.14)	4.98 (0.14)	4.69 (0.28)	1	4.18 (0.57)	4
	Dispute between WUA members	2.78 (0.58)	2.57 (0.68)	2.19 (0.6)	2.52 (0.62)	29	2.75 (0.92)	3.41 (0.64)	2.52 (0.81)	2.88 (0.8)	27	2.33 (0.62)	4.75 (0.63)	2.17 (0.85)	3.08 (0.71)	27	2.83 (0.72)	27

**Fig. 4:**
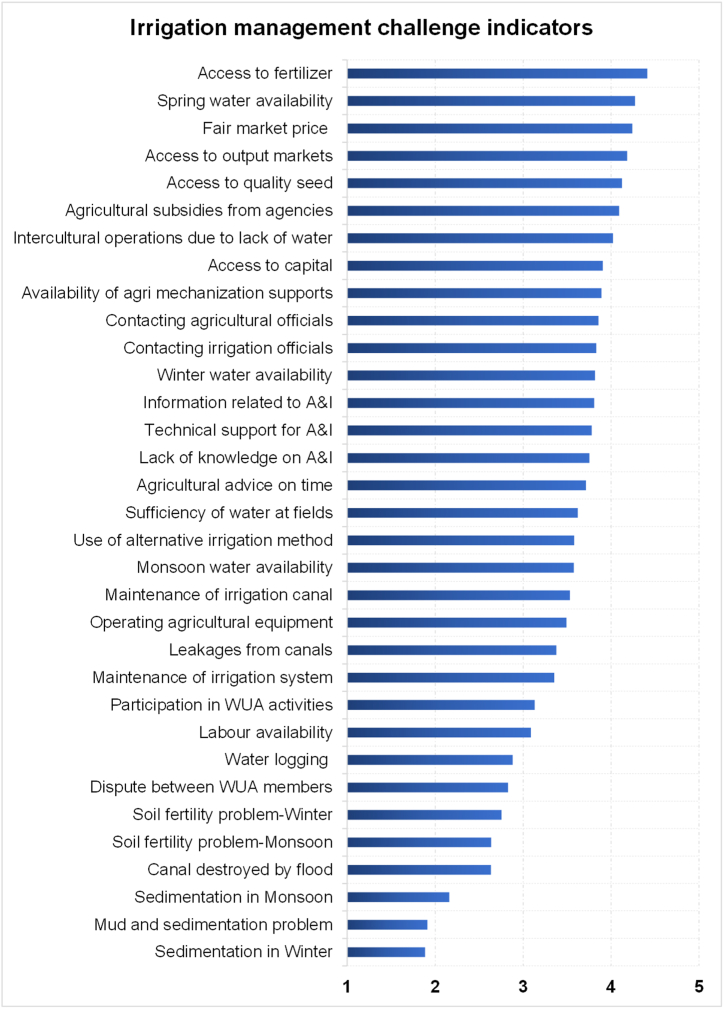
Indicators-wise combined challenge score in all 3 irrigation projects (Note: A&I – Agriculture and Irrigation, WUA- Water User Association).

Fig. 4 shows the irrigation management challenge indicators across the system including the average score for three irrigation systems. Combining all the challenges over these three irrigation systems, the top three challenges are ‘problem of timely fertilizer availability,’ ‘water availability in the spring season’ and ‘Fair market price’. The three least challenges are sedimentation in winter, sedimentation and mud problem, and sedimentation in summer (Fig. 4 and Table 11).

## 4 Discussion

The paper developed and applied a holistic assessment of irrigation and agricultural management challenges from a multi-disciplinary perspective. This paper found that the largest category of challenge across the three irrigation systems is socio-economic and market conditions, while the key specific challenges are: timely access and availability of fertilizers, spring water availability and fair market prices of agricultural products. In addition, the Babai Irrigation Project faced the most substantial challenges among the three systems, particularly in the mid section. The paper also discusses the interconnectedness of these challenges.

Socio-economic and market conditions are most critical for agricultural production.

The paper's first finding –i.e., that the largest area of challenge is socio-economic and market conditions – is broadly consistent with other literature particularly from western Nepal [29] and Nepal in general [62,63]. Nonetheless, approaches and findings varied somewhat across these documents. For example, whereas this paper relied on a spatially dispersed survey to generate findings in different segments of one canal in each of the three systems, other work relied generic survey with wider stakeholders. Related, the integrated approach utilized in this paper was more comprehensive than others, which typically only examined one of the four clusters of challenge areas. Compared to other studies, our findings spatially locate these problems in a particular irrigation system.

A major challenge in the three systems, with the highest score of 4.41 among all 33 indicators, is the timely unavailability of an adequate quantity of fertilizers from the socio-economic and market category. In 2021, the technical demand for major fertilizers - Urea, Diammonium Phosphate (DAP) and Muriate of Potash (MOP) - was 1.29 million metric tonnes, with 40 % (0.6 milliom metric tonnes) of it considered as effective demand at the national level [30,64]. Generally, imports from the neighbouring countries met fertilizer demands in Nepal. In recent years, there has been a surge in fertilizer demand due to intensified crop cultivation, the adoption of high-yielding varieties, and insufficient utilization of farmyard manure [38,65]. However, the supply of fertilizer is relatively steady or has even decreased. For example, only 344 thousand metric tonnes of fertilizer was supplied in 2019 and it even dropped to 227 thousand metric tonnes in 2022 [66,67]. This is exactly the period before the survey was conducted. As indicated by Gautam et al. [64], the existing fertilizer supply meets only 60 % of the effective demand with an even greater gap in western Nepal.

Our study showed that the proximity of canal systems to border areas with India affects access to fertilizers and labour availability. In the vicinity of RJKIP and BIP canal systems, farmers residing in the tail region of the canal systems close to the Indian border enjoy easier access to fertilizers from India compared to MIP. However, informal trading of fertilizers across the border has improved the fertilizer availability but also raised quality concerns. When it comes to selling agricultural products, Indian farmers receive significant subsidies for inputs, leading to lower production costs. Even when Indian products are sold at lower prices, they remain profitable, making it difficult for Nepalese farmers to compete with Indian agricultural products and get fair market prices for their products as their cost of production is higher and inputs subsidies are not common like in India.

Furthermore, the farmers rely on local input suppliers for seed and fertilizer in credit and this informal arrangement compels the farmers in BIP and RJKIP to sell their products at lower prices than the prevailing market price. In contrast, MIP faces a comparatively less severe impact from these challenges. The agricultural products produced in MIP enjoy relatively higher prices due to the demand in hilly regions, where most of their supplies are consumed. Additionally, specialized food collectors cater to these regions, creating a more favourable market scenario for MIP farmers. Overall, the location of canal and its interaction with socio-economic characteristics significantly influences the dynamics of fertilizer access, labour availability, and market access, underscoring farmers’ varying challenges in the respective regions.

GESI and governance are important enabling conditions.

GESI and governance are critical challenges in the three irrigation systems. The gender mainstreaming challenge in Nepal's water sector is also highlighted by Khadka et al. [29] and Shrestha and Clement [44]. Obtaining agricultural subsidies remains a persistent issue across systems despite diverse programs. The Government's Agricultural Development Strategy (ADS, 2015–2035) provides special subsidies for female farmers. Likewise, the Prime Minister Agriculture Modernization Project (PMAMP) and Agriculture Knowledge Center (AKC) provide subsidies to diverse groups of farmers.These initiatives extend subsidies for banana plantation, fisheries, improved seeds and agri-mechanization in the study areas (FGDs conducted in August 2022). Unfortunately, these subsidies do not specifically target smallholder farmers, complicating their access. In certain regions, accessing subsidies is comparatively easier for females than males. This trend holds for RJKIP, while in MIP and BIP, male farmers find this a greater challenge than their female counterparts.

Farmers find reaching out to agricultural and irrigation officials challenging in MIP. In both MIP and BIP, this is highlighted more by male farmers than females, indicating broader influences beyond culture. This might stem from declining male participation in agriculture. Focus group discussions revealed rising male outmigration in the area, leading to increased responsibilities for women, including contacting officials. Despite this, studies underline that women farmers encounter difficulties due to societal restrictions, household duties, and information gaps [40]. Some female farmers at Kulariya WUAs' suggested their preference for women technicians due to comfort. Further study is needed to understand why male farmers in MIP and BIP perceive this as a challenge.

Female respondents view participation in WUA activities as a more significant challenge than male respondents in all systems, despite it being the least ranked challenge. This arises from male dominance in resource management committees, resulting in policies overlooking women's needs and priorities [68,69]. Factors like workloads and socio-cultural norms limit women's involvement. Despite the Irrigation Policy 2003's, mandate for 33 % women representation in WUAs, field observations show male leadership predominance. Women occupy positions primarily to meet mandatory requirements, a practice often regarded as token participation, not functional participation. For instance, in all WUA Committees within the study areas, male members exclusively held the position of chairperson. In one particular WUA where the *Badghar* system is practised (an informal institution practised by an indigenous group, the Tharu community selects their leader to manage resources and handle disputes within the community) we even noted a case where the chairperson position had been passed down to the 4th generation male within the same family. Such male dominance in leadership positions and the socio-cultural barriers to actively participating in WUA committees have resulted in the exclusion of women from planning and decision-making processes within the WUAs.

Water availability is critical in agricultural water management.

Agricultural water management ranks as the third major challenge, with spring season water availability being the second biggest concern. Timsina et al. [70] reported that among many reasons, the low availability of water in the spring season is one of the contributing factors to not achieving the target of the 15th five-year plan (2019/20–2023/24) to increase the spring rice area of 250,000 ha. Overall, the non-availability of year-round irrigation water is the major challenge related to agricultural water management in all three systems. Cropping patterns and socioeconomic settings almost being identical in all three systems the agricultural water management challenges could be linked with the deficit at source as in BIP, inadequate structural measures as in RJKIP (ongoing construction), and insufficient effort from the concerned stakeholders for proper operation and maintenance of the system in MIP.

The water scarcity becomes particularly crucial during the dry season. Regarding BIP, its facilities can only support 25,000 ha during the summer, even though the intended area was 36,000 ha. As the design discharge of the main canal decreases from 40 cubic meters per second during the monsoon to 5 cubic meters per second in the dry season, resulting in a notable water deficit for winter irrigation.

While the other two systems benefit from perennial rivers originating from the Himalayas (Karnali for RJKIP and Mahakali for MIP), challenges still exist. RJKIP has no deficiency at the source, but it faces difficulties in effectively conveying water to the canal during the dry season due to limitations in the earthen intake. When the river level goes down during the low flow period, it causes difficulties in efficiently delivering water as the intake level ends up higher than the river level. In case of MIP, with controlled intake from the barrage area, the bilateral agreement already fixed the irrigation water supply. However, it encounters challenges in ensuring equitable and justified distribution of the available water reaching all fields due to insufficient canal structures and suboptimal operational and maintenance of the systems.

The asymmetry of the water availability between the head and the tail end of an irrigation system in Nepal has been reported in previous studies [49,[71], [72], [73]]. These differences become pronounced during winter and spring when surface water availability declines. In cases like BIP and RJKIP, the head region gets sufficient water, leaving mid and tail regions with inadequate supply. For instance, in the tail region of BIP, farmers have resorted to establishing alternative sources, like shallow boreholes, to secure water for the dry season, especially for crops like wheat. However, mid-region farmers in BIP face even greater challenges as groundwater levels continue to decline each year, becoming increasingly difficult to access (Validation meeting, 2023).

Physical and structural aspects of irrigation systems is vital for sustained irrigation flow.

Physical and structural issues in the surveyed irrigation systems posed the least concern. Sizing the canal's capacity and coverage to convey the required water in the command area posed no significant issues. However, dry season troubles, mainly in BIP's mid-section, revealed functionality of the canal structure such as frequent leakages, waterlogging, and lack of maintenance. Canal slides were observed in certain sections, causing water to flow out of fields, causing waterlogging in certain sections. In some cases, fields above canals required downstream gate blockage, straining relations and causing conflicts with downstream farmers. RJKIP faced similar problems in its mid and tail sections, underscoring the need to address structural challenges for smoother functioning and conflict reduction. Engaging stakeholders in planning and enhancing capacity during implementation could alleviate operational problems.

MIP presents distinct challenges in its canal system, particularly concerning urbanization disturbing the canal flow due to the construction of various structures and houses. Moreover, the dumping of solid wastes and plastic bottles has added to the unique challenges faced in canal management.

As observed in all irrigation systems, the physical and structural challenges can be largely attributed to the system's operation and maintenance. Being earthen systems, they face the frequent problems of canal breaching, seepage, canal slides and other damages. In BIP, these challenges are amplified due to the interruption of canal water during ongoing physical improvement and maintenance of canal systems.

Challenges are interconnected and require a holistic approach.

Our analysis shows the prevalence multiple of challenges such as biophysical, socioeconomic, market, GESI, governance and agricultural water management are strongly interconnected. Worsening one aspect leads to trade-offs and impacts overall water management. Conversely, improving one aspect can create synergies with another. For instance, poor irrigation canal infrastructure, such as higher leakages, can lead to less water being available to farms. Additionally, high variability in river flow at the intake can make it difficult for farmers to irrigate their crops year-round. Similarly, peripheral enabling conditions like timely access to seeds and fertilizers, and access to fair market prices are vital factors for farmers. While farmers consistently make efforts to manage on-farm challenges through coping and adaptation measures, they have limited control over peripheral enabling conditions, where the government needs to play a significant role. This multi-faceted nature of issues in irrigation systems highlights the need for holistic approaches from a nexus perspective to effectively address them [74].

## 5 Conclusions and policy implications

Challenges in irrigation management in Nepal have long been identified but have rarely been systematically analysed. This paper marks one of the first efforts to systematically diagnose key challenges across a set of schemes in the country. Based on our analysis, the most significant challenge is evident in BIP, followed by RJKIP and MIP. When considering different scheme regions (head, mid, and tail), the midsection of BIP presents the most pronounced issues, followed by the tail section of RJKIP. Conversely, the least problematic area is the midsection of MIP, followed by the head section of BIP. These are believed to be the most granular depiction of challenges in Nepalese irrigation schemes to date, and the approach may provide a model for the assessment of conditions on other schemes in the country.

While the approach utilized in this paper stratified challenge areas, critical linkages across these areas were apparent. Both BIP and RJKIP face year-round irrigation problems due to low water levels in the river system, yet a key underlying driver of these challenges was a socioeconomic context of rapid urbanization. Conversely, governance challenges in irrigation management between WUAs and irrigation management offices are a key barrier to agricultural production, yet an underlying driver for these challenges is triggered by insufficient water supply in water distribution systems. Additionally, market forces were key, as local farmers compete with Indian agricultural commodities in BIP and RJKIP (challenge area 4). Yet, this has not been a big issue in MIP lately, as agricultural goods met rising demand in nearby hilly areas.

While governmental and non-governmental interventions often prioritize enhancing water and agricultural productivity**,** our study also highlights the significance of peripheral enabling conditions i.e. conditions beyond the farm level, which play an equally crucial role. For instance, among the three irrigation systems, the most significant challenge identified was the timely and adequate availability of fertilizers. Related, fair market prices and access to output markets emerged as essential factors. Therefore, a comprehensive approach that addresses both on-farm challenges and peripheral enabling conditions is necessary to improve agricultural productivity effectively. The government should play a critical role in assuring peripherical enabling conditions including other on-farm challenges. The government must play a key role in addressing the peripherical enabling conditions in irrigation systems by taking holistic approaches from a nexus perspective.

Enhanced monitoring and evaluation can support design of comprehensive approaches. For example, there is no regular flow measurement in any of the irrigation systems at the main and secondary canals. Implementing regular canal flow measurement can support informed decision-making in water allocation and distribution by irrigation managers and also helps diminish conflicts between local communities, WUAs and irrigation managers.

Solutions that may emerge through monitoring may include conjunctive water management. The huge groundwater reserves in the Terai region hold significant potential to supplement surface irrigation [75,76] when the dependability of surface water from canals is uncertain. However, careful consideration must be given to the context of groundwater availability, as observed in the midsection of BIP, where access to groundwater poses challenges due to lowering groundwater levels, rendering shallow boring methods inefficient. The findings will help decision-makers and irrigation managers prioritize issues to resolve some of the challenges to enhance water and agricultural productivity in Nepal.

Government policies can be leveraged to improve agricultural production. While implementing such facilities, inclusive criteria must be set, followed, and monitored to reach the right group of farmers. The Agriculture Development Strategy (2015–2035) has also recommended replacing subsidies with a voucher system to prioritize targeted farmers and to provide them with the choice of inputs and extension services. Female applicants from the local district should be prioritised when recruiting technicians. Appointing local women as technicians will also provide synergy – helping women farmers share their problems, gain confidence, and actively lead local users' committees and networks. Women's leadership in WUAs shall be improved by promoting women leaders and capacitating them with communication, negotiation and other leadership skills. This may play a positive role in supporting GESI and improving governance in agriculture.

This paper contains two limitations mainly related to the timing of the survey and selected canal systems. For instance, problems such as access to fertilizer were noted in all irrigation systems before the survey, but the situation improved during validation meetings in July 2023. Our survey focused on representative canal systems with distinct challenges as observed in the canal structural issues in the midsection of BIP. While some challenges may be canal-specific, limitations related to GESI, governance, socioeconomic factors, and market dynamics could be relevant across all irrigation systems.

This paper provided the interdisciplinary approach for integrated assessment of irrigation and agriculture management challenges and tested the approach in three irrigation systems in western Nepal. The study resulted in a systematic and quantitative ranking of challenges into four distinct thematic categories. The flexibility of this approach allows for its application to irrigation (and other natural resources) management challenges including comparable socio-ecological settings, with the possibility of customization to suit specific contexts.

## Data availability statement

The data used in this study can be made available upon reasonable request to the corresponding author.

## CRediT authorship contribution statement

**Santosh Nepal:** Writing – review & editing, Writing – original draft, Visualization, Supervision, Methodology, Formal analysis, Data curation, Conceptualization. **Nilhari Neupane:** Writing – review & editing, Writing – original draft, Visualization, Methodology, Formal analysis, Data curation, Conceptualization. **Sanju Koirala:** Writing – review & editing, Writing – original draft, Methodology, Formal analysis, Data curation, Conceptualization. **Jonathan Lautze:** Writing – review & editing, Writing – original draft, Methodology, Conceptualization. **Ram Narayan Shrestha:** Writing – review & editing, Writing – original draft, Methodology, Formal analysis, Data curation. **Dinesh Bhatt:** Writing – review & editing, Writing – original draft, Methodology, Conceptualization. **Nirman Shrestha:** Writing – review & editing, Writing – original draft, Methodology, Formal analysis, Data curation. **Manju Adhikari:** Writing – review & editing, Data curation. **Santosh Kaini:** Writing – review & editing, Data curation. **Shanta Karki:** Writing – review & editing, Data curation. **Jigyasha Rai Yangkhurung:** Writing – review & editing, Data curation. **Kapil Gnawali:** Writing – review & editing, Data curation. **Ananta Man Singh Pradhan:** Writing – review & editing. **Krishna Timsina:** Writing – review & editing. **Saurav Pradhananga:** Writing – review & editing, Visualization, Formal analysis. **Manohara Khadka:** Writing – review & editing, Data curation, Conceptualization.

## Declaration of competing interest

The authors declare that they have no known competing financial interests or personal relationships that could have appeared to influence the work reported in this paper.

## Footnotes

1 The field visit was carried out in August and October 2022, where stakeholder consultations were carried out which included field observations, focus group discussions and key informants' interviews with WUAs, Irrigation and agricultural officials.

## Footnotes

2 A complete household roster was not readily available, and time and budget constraints did not allow for collecting a complete household roster before data collection. The number of households in each region was approximated with the information provided by the representatives of WUA of each region.
